# The Value of Combined Radial Endobronchial Ultrasound-Guided Transbronchial Lung Biopsy and Metagenomic Next-Generation Sequencing for Peripheral Pulmonary Infectious Lesions

**DOI:** 10.1155/2020/2367505

**Published:** 2020-04-06

**Authors:** Guangsheng Li, Jie Huang, Yuechuan Li, Jing Feng

**Affiliations:** ^1^Department of Respiratory and Critical Care Medicine, Tianjin Medical University General Hospital, 154 Anshan Road, Tianjin 300052, China; ^2^Department of Respiratory and Critical Care Medicine, Tianjin Chest Hospital, 261 Taierzhuang South Road, Tianjin 300222, China; ^3^Graduate School, Tianjin Medical University, Tianjin, China

## Abstract

**Background:**

Metagenomic next-generation sequencing (mNGS) is a new technology that allows for unbiased detection of pathogens. However, there are few reports on mNGS of lung biopsy tissues for pulmonary infection diagnosis. In addition, radial endobronchial ultrasound (R-EBUS) is widely used to detect peripheral pulmonary lesions (PPLs), but it is rarely used in the diagnosis of peripheral lung infection.

**Objective:**

The present study aims to evaluate the combined application of R-EBUS-guided transbronchial lung biopsy (TBLB) and mNGS for the diagnosis of peripheral pulmonary infectious lesions.

**Methods:**

From July 2018 to April 2019, 121 patients from Tianjin Medical University General Hospital diagnosed with PPLs and lung infection were enrolled in this prospective randomized study . Once the lesion was located, either TBLB or R-EBUS-guided-TBLB was performed in randomly selected patients, and mNGS was applied for pathogen detection in lung biopsy tissues. The results of mNGS were compared between the TBLB group and R-EBUS-guided TBLB group. In addition, the clinical characteristics and EBUS images from 61 patients receiving bronchoscopy for peripheral lung infectious detection were analyzed and compared with the results of mNGS.

**Results:**

The positivity rate of mNGS in R-EBUS-guided TBLB was (78.7%, 48/61) that was significantly higher than (60.0%, 36/60) in the TBLB group. Difference in the position of R-EBUS probe and image characteristics of peripheral lung infectious lesions affected the positivity rate of mNGS. Tissue collected by R-EBUS within the lesion produced higher positivity rate than samples collected adjacent to the lesion (*P*=0.030, odds ratio 17.742; 95% confidence interval, from 1.325 to 237.645). Anechoic areas and luminant areas of ultrasonic image characteristics were correlated with lower positivity rate of mNGS (respectively, *P*=0.019, odds ratio 17.878; 95% confidence interval, from 1.595 to 200.399; *P*=0.042, odds ratio 16.745; 95% confidence interval, from 1.106 to 253.479).

**Conclusions:**

R-EBUS-guided TBLB is a safe and effective technique in the diagnosis of peripheral lung infectious lesions. R-EBUS significantly facilitates the accurate insertion of bronchoscope into the lesions, which improves positivity rate of mNGS analysis in pathogen detection. The R-EBUS probe position within lesion produced a higher positivity rate of mNGS analysis. Nevertheless, the presence of anechoic and luminant areas on ultrasonic image was correlated with poor mNGS positivity rate.

## 1. Introduction

Pulmonary infection is a leading cause of death and morbidity worldwide [1]. However, accurate diagnosis of pathogen is challenging due to the complexity of respiratory tract microbiota. Hundreds of pathogens have been associated with pulmonary infections, including bacteria, virus, and fungi [2–4]. Time-consuming pathogen identification methods not only contribute to the increased morbidity and mortality of pulmonary infection, but also indiscriminate use of broad-spectrum antibiotics which impedes antimicrobial stewardship. Furthermore, the sensitivity and specificity of conventional pathogen detection methods are reduced after the introduction of antibiotic therapy. Rapid and accurate pathogen detection and identification of pulmonary infections is critical for the timely antimicrobial therapy.

Pathogen identification by mNGS is based on unbiased detection of nucleic acids isolated from clinical samples, including but not limited to tissue, body fluids, swabs, and bronchoalveolar lavage, stool or polymicrobial abscesses [5]. Applications of mNGS in infectious diseases diagnosis include lineage tracing, drug-resistance gene detection, and microbiome investigation [6–10]. However, reports on mNGS applied to lung biopsy tissues in pulmonary infection management remain scarce.

Etiology inspection in PPLs is challenging compared to central pulmonary lesions due to the limitation in obtaining histopathological specimens. Conventional bronchoscopy has difficulty in reaching the exact location of the lesions in the peripheral lung. CT-guided percutaneous biopsies are associated with a higher risk of complications including pneumothorax or hemoptysis [11, 12]. Once complicated, it is very different or even impossible to continue puncturing lung biopsy. R-EBUS-guided TBLB has been demonstrated as a safe and stable procedure and widely used in peripheral lung diseases management, particularly in peripheral lung cancer [13–19]. R-EBUS-guided TBLB is rarely applied in the diagnosis of peripheral lung infectious lesions. The aim of the current study was to investigate the value of combined R-EBUS-guided TBLB and mNGS in pathogen detection for peripheral pulmonary infectious lesions diagnosis.

## 2. Methods and Subjects

### 2.1. Subjects

A prospective randomized trial of bronchoscopic procedures was performed in Tianjin Medical University General Hospital between July 2018 and April 2019. During this period, bronchoscopy was performed in 151 patients who were suspected to have peripheral lung infection by chest spiral CT. Among these patients, 30 patients were excluded from the study due to histopathological diagnosis of lung cancer and 121 patients were eventually diagnosed as pulmonary infections. Among these patients, 61 patients were diagnosed by R-EBUS-guided TBLB and the other 60 patients by TBLB. Pathogen identification in lung biopsy tissues was performed by mNGS. Lung lesion was considered as PPL when its location was beyond the segmental bronchus [18]. The size of peripheral lung lesion was measured by the mean diameter of the lesion on the axial lung window setting from the CT images. The location and distance of each PPL from the costal and visceral pleura were also recorded. The lesion was classified as nodule or cavitation, confluent/patchy consolidation, and ground-glass opacity (GGO), according to visual assessment method based on CT attenuation and modified from a previous study [20]. The patient enrollment criteria were as follows: (I) patients with PPLs defined as abnormal growth shown detected via CT scans and diagnosed as infectious diseases and (II) age ≥18 years. The exclusion criteria were as follows: (I) age <18 years; (II) patients with history of lung surgery; and (III) patients with severe structural lung disease, cardiovascular and cerebrovascular diseases, and other reasons that cannot receive TBLB. The final diagnosis diagnosis of peripheral pulmonary infection should combine with spiral chest CT, conventional laboratory-based methods, histopathology, and mNGS results. After antimicrobial treatment, the patient's clinical symptoms were alleviated or relieved. Patients were followed up after discharge, and they had no recurrence. Spiral chest CT showed improvement or absorption of lung lesions.

### 2.2. Apparatus

Electronic video bronchoscope (Olympus BF-F260 or Olympus BF-P-260F, Olympus, Tokyo, Japan), ultrasonic host (MAJ-935, Olympus, Tokyo, Japan), R-EBUS with a 1.4 mm diameter (UM-S20-17S, Olympus, Tokyo, Japan), and Biopsy forceps (JHY-FB-18-105-O-O-A1, Changzhou Jiuhong) were used in performing R-EBUS-guided TBLB or TBLB.

## 3. Procedure

### 3.1. TBLB and R-EBUS-guided TBLB Procedure

TBLB was performed according to diagnostic flexible bronchoscopic application guide (2008 Edition) published by Chinese Medical Association Respiratory Diseases Society [21]. Bronchoscopic procedures were conducted through local anesthesia with lidocaine and intramuscular meperidine under the supervision of chest physician. No consciousness sedation was applied throughout the study. Pulse oximetry was used to monitor oxygenation during the procedure, and oxygen was administered via a nasal prong whenever required to maintain oxygen saturation >90%.

Lesions were located by studying the chest CT. The biopsy procedure was repeated until lung tissue spilled over the surface of the biopsy forceps. In the TBLB group, the operator maneuvered the bronchoscope to the suspected bronchi as far as possible until resistance was met and then conducted forceps biopsy. In the R-EBUS-guided TBLB group, the EBUS probe was inserted into the suspected bronchi through the working channel of the bronchoscope until resistance was met to detect the PPLs. Once the lesion was located, R-EBUS was performed through the working channel of the bronchoscope until resistance was met. Then, the EBUS probe was inserted into the suspected bronchi to detect the PPLs before R-EBUS-guided TBLB was performed. All pathological results were inspected by two experienced pathological doctors. To determine if iatrogenic pneumothorax had developed, initial chest radiographs were obtained 4 hrs after the procedure and follow-up chest X-rays the following day.

### 3.2. Specimen Collection and Processing

The lung biopsies were separately sent to clinical microbiology and histopathology laboratories within 2 hrs for analyses. The remaining or leftover tissue homogenates were stored at −70°C for mNGS.

The lung biopsies were cut into small pieces according to standard procedures. The 1.5 mL microcentrifuge tube with 0.7 mL lysis buffer and pieces of tissue sample and 1°g 0.5 mm glass bead were attached to a horizontal platform on a vortex mixer and agitated vigorously at 2800–3200RPM for 30 min. 0.3 mL sample was separated into a new 1.5 mL microcentrifuge tube, and DNA was extracted using the TIANamp Micro DNA Kit (DP316, TIANGEN BIOTECH) according to the manufacturer's recommendation. RNA extraction shared same tissue collection procedures as mentioned above, the extraction kit QIAamp Viral RNA Mini Kit (52904#, QIAGEN) was used for extraction of RNA, and then complementary DNA (cDNA) was generated from an RNA template by reverse transcription.

DNA libraries were constructed through DNA-fragmentation, end-repair, adapter-ligation, and PCR amplification. Agilent 2100 Bioanalyzer (Agilent Technologies, Santa Clara, CA) was used for quality control of DNA libraries. Quality qualified libraries were sequenced by BGISEQ-50 platform [22]. At least 20M reads were obtained for each sample. High-quality sequencing data were generated by removing low-quality and short (length <35 bp) reads, followed by computational subtraction of human host sequences mapped to the human reference genome (hg19) using Burrows–Wheeler Alignment [23]. The remaining data by removal of low-complexity reads were classified by simultaneously aligning to four microbial genome databases, consisting of viruses, bacteria, fungi, and parasites. The classification reference databases were downloaded from NCBI (ftp://ftp.ncbi.nlm.nih.gov/genomes/). RefSeq contains 4,061 whole genome sequences of viral taxa, 2,473 bacterial genomes or scaffolds, 199 fungi related to human infection, and 135 parasites associated with human diseases. Combining the results of controls and calibrators, data-analytical algorithms were used to exclude microorganisms that were not significantly related to infection. Microorganisms with clinical significance were reported with the sequencing reads of the micro-organisms detected at the genus/species levels.

### 3.3. Evaluation of mNGS Results in Lung Biopsy Tissues

The results of the mNGS-based approach were estimated by 2 independent clinical specialists not associated with the study.

### 3.4. Statistical Analysis

SPSS (version 17.0, SPSS Inc, Chicago, Illinois) was used for statistical analyses. The data are expressed as mean (standard deviation). Paired *t*-tests were used to compare the means of the independent variables. Pearson's chi-square test or Fisher's exact test was used for categorical variable. Multivariate logistic regression tests were used to further confirm the results of independent variable analysis. *P* value <0.05 was considered as statistically significant.

## 4. Results

### 4.1. Study Population

A total of 121 patients with peripheral lung infectious lesions were enrolled in a prospective randomized study, 60 patients in the TBLB group and 61 patients in the R-EBUS-guided TBLB group. Their baseline characteristics are shown in Table 1. No significant difference in baseline characteristics was observed between the TBLB group and R-EBUS-guided TBLB group, *P* > 0.05.

### 4.2. Diagnostic Yield of mNGS

Final diagnosis of targeted lesions is shown in Table 2. In the TBLB group, bacteria infection was identified in 30 patients (50%), virus infection in 12 patients (20%), fungal infection in 18 patients (30%), atypical pathogen infection in 6 patients (10%), and mycobacterium tuberculosis (MTBC) infection in 9 patients (15%). In the R-EBUS-guided TBLB group, bacteria infection was identified in 33 patients (54.1%), virus infection in 11 patients (18%), fungal infection in 21 patients (34.4%), atypical pathogen infection in 7 patients (11.5%), and MTBC infection in 10 patients (16.4%). mNGS successfully identiﬁed the pathogens in 48 out of 61 patients (78.7%) in the R-EBUS-guided TBLB group, and 36 out of 60 patients (60.0%) in the TBLB Group. There was significant difference in the positivity rate between the R-EBUS-TBLB group and the TBLB group (78.7%/60.0%, *P*=0.026).

### 4.3. The Most Commonly Identified Pathogens by mNGS in Immunocompromised and Non-Immunocompromised Patients

More than half of the 121 enrolled patients had hematological malignancies, including 70 immunocompromised and 51 immunocompetent individuals. Immunocompromised individuals were more susceptible to lung infections than the immunocompetent ones. The most common pathogens detected by mNGS are shown in Table 3.

### 4.4. Relationship Between Lesions Characteristics and Diagnostic Yield of mNGS in the R-EBUS Group

In the R-EBUS group, the diameter of lesion region >3 cm was associated with significantly better diagnostic yield by univariate analysis (Table 2, *P*=0.013, odds ratio 6.080; 95% confidence interval (CI), from 1.630 to 22.685). However, the significance was lost after multivariate analysis combined with the R-EBUS probe position and EBUS images characteristics (Table 2, *P*=0.067, odds ratio 8.757; 95% confidence interval (CI), from 0.858 to 89.417). Distance from the chest wall of the lesion was not associated with the diagnostic yield of mNGS (Table 2, *P*=0.057). The presence of probe within the lesion were associated with significantly better diagnostic yield with radial EBUS than when the probe was found adjacent to the lesion (Table 2, *P*=0.030, odds ratio 17.742; 95% confidence interval (CI), from 1.325 to 237.645).

**Figure 1:**
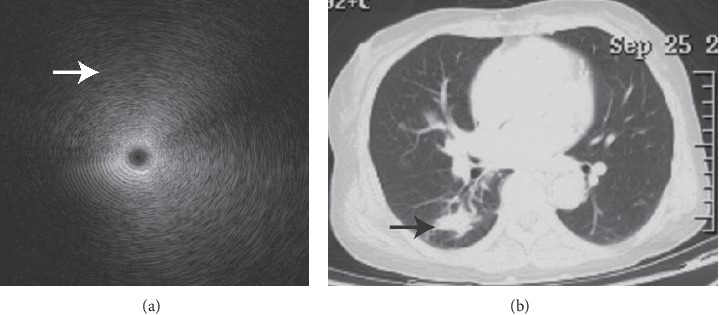
Homogenous internal echoes. Image of a 72-year-old woman with a peripheral consolidation lesion over the right lower lung lobe by chest radiography ((b) black arrow) with parrot chlamydia pneumonia identified by mNGS. EBUS demonstrated homogeneous internal echoes without margins (a). The particles displayed a formation of concentric circles around the echo probe, and entire images exhibited a sense of gradation. The particles lengthened to form a very short arc of the circumference, particularly in the outer part (white arrow).

**Figure 2:**
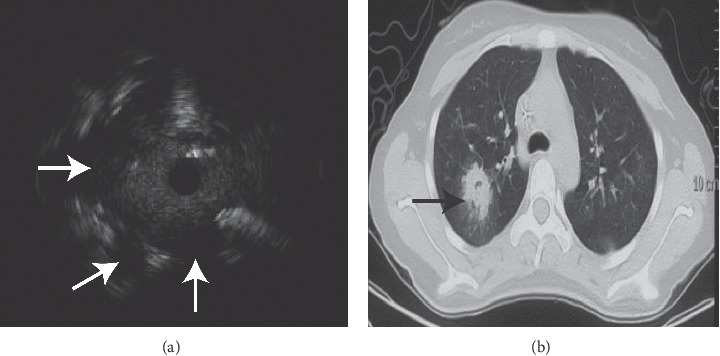
EBUS demonstrated heterogeneous internal echoes, anechoic areas, and luminant areas (a). There were three anechoic areas ((a) white arrows) in the image from a 21-year-old female with a peripheral lesion over the right upper lobe with *Àspergillus* infection identified by mNGS ((b) black arrow).

**Figure 3:**
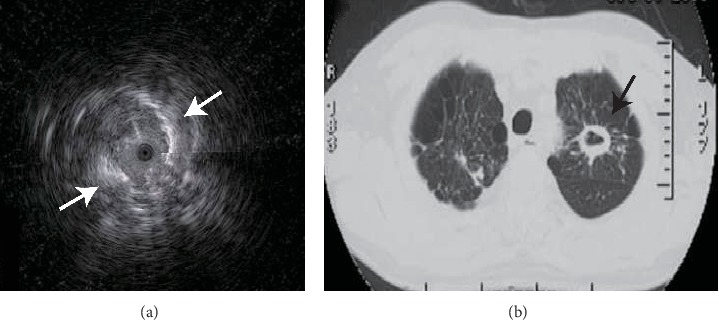
An EBUS image of pulmonary tuberculosis. The image was from a 55-year-old man with a left upper lobe cavity lesion ((b) black arrow). The image contained heterogeneous internal echoes and luminant areas ((a) white arrow).

**Figure 4:**
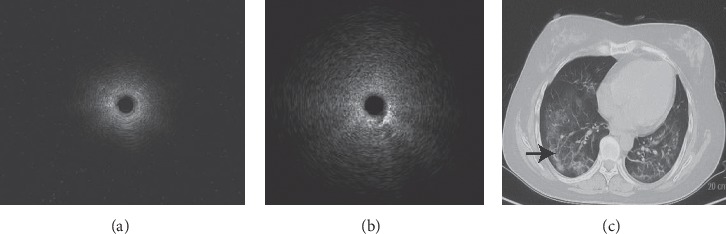
The EBUS image of normal lung parenchyma surrounding bronchial structures had patchy and numerous hyperechoic particles that were typically called snowstorm-like. The image represented normal air-filled alveoli (a), an EBUS image of Pneumocystis carinii pneumonia (PCP). The image was from a 46-year-old woman which demonstrates mixed blizzard sign, the internal echo of the lesions demonstrated diffuse heterogeneity with several hyperechoic dots and linear arcs that were distributed irregularly (b). The CT manifestations included bilateral diffuse ground-glass opacity (c). Biopsy was performed at part-solid GGO lesion in the right lower lung lobe, the diagnosis of pneumocystis carinii infection identified by mNGS ((c) black arrow).

The EBUS image of normal lung parenchyma surrounding bronchial structures had patchy and numerous hyperechoic particles that were typically called snowstorm-like [24]. The particles of homogeneous internal echoes pictures were unanimous in size, echogenicity, and distribution, and the echogenicity was invariably slightly lower than that in normal lung parenchyma [24]. The major lesions with homogeneous internal echoes were pneumonia, characterized by exudate-filled alveoli [25] (Figure 1). The heterogeneous internal echoes displayed a mosaic pattern in imaging particle distribution, and the particles varied in size. Image features presented echo-free areas consistent with areas of necrosis histopathologically [26] (Figure 2). Luminant areas looked like a fusion of enormous sparkled dots of varying size and shape, which may be the result of bronchus destruction, condensed air within necrotic areas, or calcification (Figure 3). Non-luminant areas were likely to be nonneoplastic [25]. The EBUS images of GGO lesions included pure and part-solid GGO, which named blizzard or mixed blizzard are often malignant. The pure type usually demonstrates a subtle but noticeable increase in the intensity and radius of the whitish acoustic shadow of normal lung on EBUS. In the mixed blizzard sign (Figure 4), the internal echo of the lesions demonstrated diffuse heterogeneity with several hyperechoic dots, linear arcs, and vessels that were distributed irregularly or combined with the blizzard sign [27, 28].

In our study, pathogens were successfully detected by mNGS mostly in the lesion with homogeneous internal echoes (35 of 48 cases, 72.9%), and 4 lesions (4 of 13 cases, 30.8%) with homogeneous internal echoes were negative in mNGS analysis. More than half of cases exhibited the characteristics of anechoic area in mNGS negative cases (8 of 13 cases, 61.5%), while only 5 cases had positive mNGS results with anechoic areas, included 1 case of pulmonary tuberculosis and 1 lung abscess. There were 8 cases involving luminant areas with positive mNGS results (8 of 48 cases, 16.7%), and 8 cases with negative mNGS (8 of 13 cases, 38.5%). Homogenous internal echoes were correlated with better diagnostic yield of mNGS from lung biopsy tissues by univariate analysis (Table 2, *P*=0.005; odds ratio 6.058; 95% confidence interval (CI), 1.588 to 23.107). However, the significance was lost after multivariate analysis combined with the R-EBUS probe position and EBUS images characteristics (Table 2, *P*=0.061, odds ratio 8.598; 95% confidence interval (CI), from 0.903 to 81.885). Anechoic areas and luminant areas of ultrasonic image characteristics were correlated with lower diagnostic yield of mNGS (Table 2, respectively, *P*=0.019, odds ratio 17.878; 95% confidence interval (CI), from 1.595 to 200.399; *P*=0.042, odds ratio 16.745; 95% confidence interval (CI), from 1.106 to 253.479).

### 4.5. Complications

Over all, complications related to TBLB group occurred in 4 patients (6.6%): mild bleeding was developed in 2 patients (3.3%) and pneumothorax was developed in 2 patients (3.3%). One patient (1.6%) developed mild bleeding, and one patient (1.6%) developed pneumothorax in the R-EBUS-guided TBLB group. The bleeding was improved after local application of norepinephrine and intravenous drip of vasopressin. The pneumothorax was resolved spontaneously without chest tube drainage. No patient suffered from severe hemorrhage, air embolism, respiratory failure, or pulmonary infection after the procedure. There were no premature terminations of the procedure, and none of the patients died due to application of the procedure. No significant differences in the occurrence of complications were detected between the TBLB group and R-EBUS-guided TBLB group (Table 1, *P* > 0.05).

## 5. Discussion

Lack of accurate etiology diagnosis remains the major cause of high morbidity and mortality in pulmonary infections, which increases healthcare expense. mNGS is an unbiased and rapid technique capable of detecting a broad range of pathogenic bacterial, viral, fungal, and parasite simultaneously in clinical samples. R-EBUS is a safe and valuable technique, which has been demonstrated to increase diagnostic yield for PPLs [29]. Current clinical use of R-EBUS is mainly in the localization of peripheral pulmonary lesions prior to biopsy. Reports on mNGS in pulmonary infection diagnosis with lung biopsy tissues remain scarce, and there are no reports of combined peripheral ultrasound for peripheral pulmonary infectious diseases diagnosis [30–32]. Li et al. applied mNGS to detect the presence of pathogenic microbes in lung biopsy tissues through lung puncture, and the results showed that the diagnostic yield of mNGS was 75% (15 of 20 cases) [33]. Multiple studies have shown that EBUS helps to accurately locate PPL region and improves the diagnostic accuracy of transbronchial biopsy (TBB) [25, 34–36]. In this study, mNGS successfully identified pathogens in 48 out of 61 patients in the R-EBUS-TBLB group, and 37 out of 60 patients in the TBLB group. There was significant difference in the diagnostic yield between two groups (78.7%/60.0%, *P* < 0.05). Previous studies reported that the diagnostic yields of utilize EBUS with a guide sheath or EBUS-guided biopsy in PPL were 53–75.9% [13–18, 37]. Our study produces better diagnostic yields than previous studies. The results suggest that R-EBUS is useful in confirming the accurate insertion of the bronchoscope into lesions and could improve the positivity rate of mNGS in pathogen detection.

This study showed that the diagnostic yield of pathogens detection by mNGS with TBLB was not related to the size of lesion and its distance to the chest wall of peripheral infection lesions; this is not consistent with previous studies [13–18, 33, 36]. The reason may be that we did not have sufficient sample size. In our study, the presence of probe within the lesion was associated with significantly better diagnostic yield with R-EBUS than that when the probe was found adjacent to the lesion, which is consistent with previous studies [13–18, 33, 36]. These findings suggest that when the probe was in the center of the lesion, there was a transmural exudation and invasion of the bronchus by the infection than when it was adjacent to the lesion.

Our study indicated that ultrasound image characteristics of peripheral lung infectious lesions were correlated with the diagnostic yield of mNGS. The image of anechoic areas and luminant areas of ultrasonic image characteristics were correlated with worse diagnostic yield of mNGS from lung biopsy tissues. Lesions with anechoic areas and luminant areas might result from necrosis, where airway inflammation is light, pathogen load is low, and lesions tend to be chronic. Therefore, the diagnostic yield of mNGS approaches after TBLB is low.

There were several limitations to our study. First, it was conducted at a single institute owing to the difficulties in recruiting subjects, so there was not sufficient sample size. Second, the majority of patients had hematological malignancy, and potential selection bias might influence the results. Third, we did not use fluoroscopy, electromagnetic navigation bronchoscopy, or virtual bronchoscopy with R-EBUS, all of which could have further improved the diagnostic accuracy of radial EBUS-guided biopsy. Considering the medical expenses, the enrolled patients did not receive mNGS analysis of bronchoalveolar lavage fluid or serology at the same time. Whether lung tissue mNGS is superior to bronchoalveolar lavage fluid or serology in patients with pulmonary infection required further investigation. Furthermore, studies designed as multicenter trials are needed to validate the result from this study.

## 6. Conclusion

Pneumonia is a common infection that often lacks pathogen diagnosis. Rapid and accurate pathogen detection and identification of pathogen is critical for the precise antibiotic therapy. Many patients are on antibiotic therapy, which limits the yield of culture-based testing. Thus, we hope the early use of mNGS testing to identify the pathogen in the everyday clinical practice. For PPLs, we can combine with R-EBUS. Considering the high cost of mNGS test, we recommend the early use of mNGS testing in failure cases of empirical treatment and severe cases.

Although there are some limitations, our study firstly demonstrated that pathogens diagnosis of peripheral lung infectious lesions by mNGS in TBLB was meaningful and that R-EBUS-guided TBLB is useful in confirming the accurate insertion of the bronchoscope into PPLs, which improved diagnostic yield of mNGS. The R-EBUS probe position within the lesion was correlated with higher diagnostic yield of mNGS from lung biopsy tissues. EBUS patterns of peripheral pulmonary lesions with anechoic areas and luminant areas were correlated with the diagnostic yield of mNGS.

## Figures and Tables

**Table 1:** Baseline characteristics of study population^*∗*^.

Groups	TBLB group	R-EBUS-TBLB group	t/*χ*2 value	*P* value
Particients	60	61		
Age ± 2SD (years)	55.8 ± 12.5	55.4 ± 11.5	1.168	0.243
Male/female	38/22	36/25	0.237	0.626
Immunodeficiency				
Hematological malignancy	34 (56.7)	36 (59.0)	0.068	0.794
Lesion location *n*%				
Lower lobe	27 (45.0)	33 (54.1)	2.524	0.283
Right upper lobe	16 (26.7)	18 (29.5)		
Left upper lobe	17 (28.3)	10 (16.4)		
Leision size				
≤3 cm	19 (31.7)	18 (29.5)	0.066	0.797
>3 cm	41 (68.3)	43 (70.5)		
Distance from the chest wall				
≤3 cm	22 (36.7)	28 (45.9)	1.064	0.302
>3 cm	38 (63.3)	33 (54.1)		
CT findings				
Nodule or cavitation	19 (31.7)	28 (45.9)	2.720	0.257
Confluent or patchy consolidation	29 (48.3)	22 (36.1)		
GGO	12 (20.0)	11 (18.0)		
Complications				
Mild bleeding	2 (3.3)	1 (1.6)	0.359	0.549
Pneumothorax	2 (3.3)	1 (1.6)	0.356	0.551
Final diagnosis				
Bacteria (mycobacteria excluded)	30 (50)	33 (54.1)		
Virus	12 (20)	11 (18)		
Fungus	18 (30)	21 (34.4)		
Atypical pathogen	6 (10)	7 (11.5)		
MTBC	9 (15)	10 (16.4)		
Positivity of mNGS	36 (60.0)	48 (78.7)	4.977	0.026

GGO, ground-glass opacity. MTBC, mycobacterium tuberculosis complex. ^*∗*^Data are presented as no. (%) or mean ± SD unless otherwise indicated.

**Table 2:** The relationship between clinical and image characteristics of PPLs and the diagnostic yield of R-EBUS-guided TBLB detected infectious pathogens by mNGS^*∗*^.

Variables	mNGS diagnostic	mNGS nondiagnostic	Univariate	Multivariate
			OR *P* value	OR *P* value
Lesion size				
>3 cm	38 (79.2)	5 (38.5)	6.080 0.013	8.757 0.067
≤3 cm	10 (20.1)	8 (61.5)		
Distance from the chest wall				
>3 cm	29 (60.4)	4 (30.8)	3.434 0.057	
≤3 cm	19 (39.6)	9 (69.2)		
R-EBUS probe position				
Within	34 (70.8)	4 (30.8)	5.464 0.009	17.742 0.030
Adjacent to	14 (29.2)	9 (69.2)		
Internal echoes				
Homogeneity	35 (72.9)	4 (30.8)	6.058 0.005	8.598 0.061
Heterogeneity	13 (27.1)	9 (69.2)		
Anechoic areas				
Yes	5 (10.4)	8 (61.5)	13.760 < 0.001	17.878 0.019
No	43 (89.6)	5 (38.5)		
Luminant areas				
Yes	8 (16.7)	8 (61.5)	8 0.001	16.745 0.042
No	40 (83.3)	5 (38.5)		
Total cases	48	13		

PPLs, peripheral lung lesions. R-EBUS, radial endobronchial ultrasound. TBLB, transbronchial lung biopsy. mNGS, metagenomic next-generation sequencing. ^*∗*^Data are presented as no. (%) unless otherwise indicated.

**Table 3:** The most commonly identified pathogens by mNGS in 121 patients^*∗*^.

mNGS results	Immunocompromised (*n* = 70)	Nonimmunocompromised (*n* = 51)
Bacteria	*Pseudomonas aeruginosa* 9 (12.9)	*Pseudomonas aeruginosa* 4 (7.8)
	*Klebsiella pneumoniae* 7 (10.0)	*Klebsiella pneumoniae* 3 (5.9)
	*Acinetobacter baumannii* 6 (8.6)	*Acinetobacter baumannii* 3 (5.9)
	*MTBC* 6 (8.6)	*MTBC* 8 (15.7)

Viruses	*EBV* 11 (15.7)	*EBV* 4 (7.8)
	*CMV* 8 (11.4)	*CMV* 4 (7.8)
	*Circovirus* 5 (7.1)	*Circovirus* 3 (5.9)

Fungus	*Aspergillus* 11 (15.7)	*Aspergillus* 4 (7.8)
	*Pneumocystis carinii* 7 (10.0)	*Pneumocystis carinii* 3 (5.8)
	*Rhizopus carinii* 4 (5.7)	*Actinomyces* 2 (3.9)

Atypical pathogen	*Mycoplasma* 4 (5.7)	*Mycoplasma* 2 (3.9)
	*Chlamydia* 2 (2.9)	*Chlamydia* 1 (2.0)
		*Legionellal* 1 (2.0)

mNGS, metagenomic next-generation sequencing. MTBC, mycobacterium tuberculosis complex. EBV, Epstein–Barr virus. CMV, cytomegalovirus. ^*∗*^Data are presented as no. (%) unless otherwise indicated.

## Data Availability

All data are fully available without restriction.

## References

[1] Magill S. S., Edwards J. R., Bamberg W. (2014). Multistate point-prevalence survey of health care-associated infections. *New England Journal of Medicine*.

[2] Renaud C., Campbell A. P. (2011). Changing epidemiology of respiratory viral infections in hematopoietic cell transplant recipients and solid organ transplant recipients. *Current Opinion in Infectious Diseases*.

[3] Ruppé E., Baud D., Schicklin S., Guigon G., Schrenzel J. (2016). Clinical metagenomics for the management of hospital- and healthcare-acquired pneumonia. *Future Microbiology*.

[4] De La Cruz O., Silveira F. P. (2017). Respiratory fungal infections in solid organ and hematopoietic stem cell transplantation. *Clinics in Chest Medicine*.

[5] Gu W., Miller S., Chiu C. Y. (2019). Clinical metagenomic next-generation sequencing for pathogen detection. *Annual Review of Pathology: Mechanisms of Disease*.

[6] Gardy J. L., Loman N. J. (2018). Towards a genomics-informed, real-time, global pathogen surveillance system. *Nature Reviews Genetics*.

[7] Wang C., Mitsuya Y., Gharizadeh B., Ronaghi M., Shafer R. W. (2007). Characterization of mutation spectra with ultra-deep pyrosequencing: application to HIV-1 drug resistance. *Genome Research*.

[8] Lefterova M. I., Suarez C. J., Banaei N., Pinsky B. A. (2015). Next-generation sequencing for infectious disease diagnosis and management. *The Journal of Molecular Diagnostics*.

[9] Sahoo M. K., Lefterova M. I., Yamamoto F. (2013). Detection of cytomegalovirus drug resistance mutations by next-generation sequencing. *Journal of Clinical Microbiology*.

[10] Weinstock G. M. (2012). Genomic approaches to studying the human microbiota. *Nature*.

[11] Boskovic T., Stojanovic M., Stanic J. (2014). Pneumothorax after transbronchial needle biopsy. *Journal of Thoracic Disease*.

[12] Boskovic T., Stanic J., Pena-Karan S. (2014). Pneumothorax after transthoracic needle biopsy of lung lesions under CT guidance. *Journal of Thoracic Disease*.

[13] Fuso L., Varone F., Magnini D. (2013). Role of ultrasound-guided transbronchial biopsy in the diagnosis of peripheral pulmonary lesions. *Lung Cancer*.

[14] Huang C.-T., Ho C.-C., Tsai Y.-J., Yu C.-J., Yang P.-C. (2009). Factors influencing visibility and diagnostic yield of transbronchial biopsy using endobronchial ultrasound in peripheral pulmonary lesions. *Respirology*.

[15] Minezawa T., Okamura T., Yatsuya H. (2015). Bronchus sign on thin-section computed tomography is a powerful predictive factor for successful transbronchial biopsy using endobronchial ultrasound with a guide sheath for small peripheral lung lesions: a retrospective observational study. *BMC Medical Imaging*.

[16] Chavez C., Sasada S., Izumo T. (2015). Endobronchial ultrasound with a guide sheath for small malignant pulmonary nodules: a retrospective comparison between central and peripheral locations. *Journal of Thoracic Disease*.

[17] Durakovic A., Andersen H., Christiansen A., Hammen I. (2015). Retrospective analysis of radial EBUS outcome for the diagnosis of peripheral pulmonary lesion: sensitivity and complications. *European Clinical Respiratory Journal*.

[18] Yamada N., Yamazaki K., Kurimoto N. (2007). Factors related to diagnostic yield of transbronchial biopsy using endobronchial ultrasonography with a guide sheath in small peripheral pulmonary lesions. *Chest*.

[19] Ost D. E., Ernst A., Lei X. (2016). Diagnostic yield and complications of bronchoscopy for peripheral lung lesions. Results of the AQuIRE registry. *American Journal of Respiratory and Critical Care Medicine*.

[20] Luigia R., Antonio P., Stefanella M. (2008). Intensive-care unit lung infections:The role of imaing with special empasis on multi-detector row computed tomography. *European Journal of Radiology*.

[21] Chinese Medical Association (2008). TBLB according to the Chinese medical association respiratory diseases society, “diagnostic flexible bronchoscopic application guide (2008). *Chinese Journal of Tuberculosis and Respiratory Diseases*.

[22] Jeon Y. J., Zhou Y. L., Li Y. H. (2014). The fesibility study of non-invasive fetal trisomy 18 and 21 detection with semiconductor sequencing platform. *PLoS One*.

[23] Li H., Durbin R. (2009). Fast and accurate short read alignment with Burrows-Wheeler transform. *Bioinformatics*.

[24] Chao T.-Y., Lie C.-H., Chung Y.-H., Wang J.-L., Wang Y.-H., Lin M.-C. (2006). Differentiating peripheral pulmonary lesions based on images of endobronchial ultrasonography. *Chest*.

[25] Kurimoto N., Murayama M., Yoshioka S., Nishisaka T. (2002). Analysis of the Internal Structure of Peripheral Pulmonary Lesions Using Endobronchial Ultrasonography. *Chest*.

[26] Lie C.-H., Chao T.-Y., Chung Y.-H., Wang J. L., Wang Y.-H., Lin M.-C. (2009). New image characteristics in endobronchial ultrasonography for differentiating peripheral pulmonary lesions. *Ultrasound in Medicine & Biology*.

[27] Sasada S., Izumo T., Chavez C., Tsuchida T. (2014). Blizzard Sign as a specific endobronchial ultrasound image for ground glass opacity: a case report. *Respiratory Medicine Case Reports*.

[28] Izumo T., Sasada S., Chavez C. (2015). Radial endobronchial ultrasound images for ground-glass opacity pulmonary lesions. *European Respiratory Journal*.

[29] Huang C.-T., Tsai Y.-J., Chi Ho C.-, Chong-Jen Y. (2017). The value of repeat radial-probe endobronchial ultrasound-guided transbronchial biopsy after initial nondiagnostic results in patients with peripheral pulmonary lesions. *BMC Pulmonary Medicine*.

[30] Langelier C., Zinter M. S., Kalantar K., Yanik G. A., Christenson S. (2017). Metagenomic sequencing detects respiratory pathogens in hematopoietic cellular transplant patients. *American Journal of Respiratory and Critical Care Medicine*.

[31] Pendleton K. M., Erb-Downward J. R., Bao Y. (2017). Rapid pathogen identification in bacterial pneumonia using real-time metagenomics. *American Journal of Respiratory and Critical Care Medicine*.

[32] Graf E. H., Simmon K. E., Tardif K. D. (2016). Unbiased detection of respiratory viruses by use of RNA sequencing-based metagenomics: a systematic comparison to a commercial PCR panel. *Journal of Clinical Microbiology*.

[33] Li H., Gao H., Han M., Wang Qi (2018). Detection of pulmonary infectious pathogens from lung biopsy tissues by metagenomic next-generation sequencing. *Frontiers in Cellular and Infection Microbiology*.

[34] Herth F. J. F., Ernst A., Becker H. D. (2002). Endobronchial ultrasound-guided transbronchial lung biopsy in solitary pulmonary nodules and peripheral lesions. *European Respiratory Journal*.

[35] Kurimoto N., Miyazawa T. (2004). Endobronchial ultrasonography. *Seminars in Respiratory and Critical Care Medicine*.

[36] Chung Y.-H., Lie C.-H., Chao T.-Y. (2007). Endobronchial ultrasonography with distance for peripheral pulmonary lesions. *Respiratory Medicine*.

[37] Su J. Z., Ming Z., Jun Z. (2016). Comparison of radial endobronchial ultrasound with a guide sheath and with distance by thin bronchoscopy for the diagnosis of peripheral pulmonary lesions: a prospective randomized crossover trial. *Journal of Thoracic Disease*.

